# Quantitative EEG parameters correlate with the progression of human prion diseases

**DOI:** 10.1136/jnnp-2016-313501

**Published:** 2016-07-13

**Authors:** Edit Franko, Tim Wehner, Olivier Joly, Jessica Lowe, Marie-Claire Porter, Joanna Kenny, Andrew Thompson, Peter Rudge, John Collinge, Simon Mead

**Affiliations:** 1 NHS National Prion Clinic, National Hospital for Neurology and Neurosurgery, University College London Hospitals NHS Foundation Trust, London, UK; 2 MRC Prion Unit, Department of Neurodegenerative Disease, UCL Institute of Neurology, London, UK; 3 Department of Clinical Neurophysiology, National Hospital for Neurology and Neurosurgery, University College London Hospitals NHS Foundation Trust, London, UK; 4 MRC Cognition and Brain Sciences Unit, Cambridge, UK

## Abstract

**Background:**

Prion diseases are universally fatal and often rapidly progressive neurodegenerative diseases. EEG has long been used in the diagnosis of sporadic Creutzfeldt-Jakob disease; however, the characteristic waveforms do not occur in all types of prion diseases. Here, we re-evaluate the utility of EEG by focusing on the development of biomarkers. We test whether abnormal quantitative EEG parameters can be used to measure disease progression in prion diseases or predict disease onset in healthy individuals at risk of disease.

**Methods:**

In the National Prion Monitoring Cohort study, we did quantitative encephalography on 301 occasions in 29 healthy controls and 67 patients with prion disease. The patients had either inherited prion disease or sporadic Creutzfeldt-Jakob disease. We computed the main background frequency, the α and θ power and the α/θ power ratio, then averaged these within 5 electrode groups. These measurements were then compared among participant groups and correlated with functional and cognitive scores cross-sectionally and longitudinally.

**Results:**

We found lower main background frequency, α power and α/θ power ratio and higher θ power in patients compared to control participants. The main background frequency, the power in the α band and the α/θ power ratio also differed in a consistent way among the patient groups. Moreover, the main background frequency and the α/θ power ratio correlated significantly with functional and cognitive scores. Longitudinally, change in these parameters also showed significant correlation with the change in clinical and cognitive scores.

**Conclusions:**

Our findings support the use of quantitative EEG to follow the progression of prion disease, with potential to help evaluate the treatment effects in future clinical-trials.

## Introduction

Prion diseases are fatal, progressive, neurodegenerative diseases caused by accumulation of an abnormal isoform of the cellular prion protein in and around neurons leading to synaptic dysfunction and eventual neuronal loss.^1^ The most common type is sporadic Creutzfeldt-Jakob disease (sCJD) which accounts for ∼85% of the annual incidence of prion disease. There are strong genetic susceptibility factors in prion disease, with ∼15% of cases being an inherited disease, all caused by mutation of the prion protein gene (*PRNP*).^2^ A small proportion is acquired, caused by the transmission of prions during blood transfusion, neurosurgery, via pituitary-derived growth hormone therapy or ingestion of bovine spongiform encephalopathy-contaminated food products.^1^

Based on well-established epidemiological criteria, the diagnosis of sCJD is termed ‘possible’ if the patient has progressive dementia with <2 years duration accompanied with at least two of the following clinical features: myoclonus, visual or cerebellar disturbance, pyramidal or extrapyramidal dysfunction and akinetic mutism. EEG recording can increase the confidence of the diagnosis from ‘possible’ to ‘probable’ sCJD if generalised triphasic periodic sharp wave complexes (PSWCs) are demonstrated. PSWCs have been reported in many studies;^3–6^ they appear relatively late after symptom onset and are usually accompanied by movement disorders (eg, myoclonus, dyskinesia and startle) and reduced responsiveness to stimuli (akinetic mutism or coma).^7^ ^8^ Moreover, PSWCs are not equally present in the different subtypes of sCJD.^9^ Other biomarkers are available for the diagnosis of prion disease, notably signal change in the basal ganglia, thalamus and cortex on diffusion-weighted MRI, and assays of abnormal prion protein or its seeding activity in tissue or biofluids.^10–13^

Several experimental therapeutics have shown promise in animal prion diseases and are being developed with a view to human studies.^14–16^ Therefore there is an unmet need for biomarkers, which have utility for experimental medicine: those, which provide objective evidence of disease progression in symptomatic patients, and which predict clinical onset in individuals at-risk of disease because they have been exposed to prions or carry a *PRNP* mutation.

A potentially relevant approach is quantitative EEG (qEEG) analysis. This extends the pure visual inspection of the EEG recordings with more precise and objective measurements. They might therefore allow the clinician to detect subtle changes in the EEG much before they would be visible to the naked eye and before PSWCs appear. qEEG techniques are more sensitive markers of the progression in other diseases and can show stronger correlation with the clinical signs.^17^ ^18^

Here, for the first time, we applied qEEG analysis cross-sectionally and longitudinally in 67 patients with or at-risk of prion disease in the context of a national prospective observational cohort study, and compared them to healthy controls. Our aim was to evaluate the potential of qEEG in prion disease as an objective biomarker of (1) disease progression and (2) prediction of disease onset in healthy individuals. To this end, we automatically and quantitatively characterised the EEG changes before the occurrence of the typical PSWCs and correlated these measurements with newly developed functional measures (MRC Prion Disease Rating Scale) and cognitive function (Mini Mental State Examination (MMSE)) of the patients. Having repeated recordings from patients, we also evaluated a correlation between change in qEEG parameters and change in functional and cognitive abilities.

## Materials and methods

### Participants

Participants were recruited to the National Prion Monitoring Cohort (2008–2013).^19^ This study aims to collect longitudinal, prospective, systematic, clinical history and examination, rating scale and functional data from patients with prion disease and their relatives who have increased risk of developing the disease. A key achievement of the Cohort study to date is the development of a bespoke functionally orientated rating scale (the MRC Prion Disease Rating Scale, subsequently termed the MRC Scale). We selected those participants who had an EEG recorded at the National Hospital for Neurology and Neurosurgery (NHNN). The criteria for exclusion were that the whole EEG recording was degraded by artefacts (leaving <40 artefact-free epochs) or had a very small amplitude signal. The number of participants excluded from the analysis is shown in table 1.

**Table 1:** Demographic data

	n (excluded)	Male	Age in years (mean±SD)	Range	MRC score at baseline	MMSE at baseline	Number of participants with repeat data (max. number of repeats)	Time between first and last EEG in days (mean±SD)	Time between symptom onset and first EEG in days (mean±SD)
Controls	29 (2)	17	49.2±12	24–69	20	29.51±0.9	28 (3)	539.0±194.7	NA
Patients	67 (6)	32	47.3±13.7	20–81	17.03±4.7	22.98±8.33	44 (6)	641.0±369.4	NA
Asymptomatic IPD	23 (2)	9	40±13.7	20–72	20	29±1.52	16 (5)	837.8±333.5	NA
Symptomatic IPD	30 (0)	14	46.2±10.2	26–70	16.98±4.13	20.82±8.81	25 (6)	582.8±325.2	1318±1756
sCJD	14 (4)	9	61.8±9.2	44–81	11.18±5.6	16.3±7.7	3 (3)	78±61.4	299±191

The baseline MRC Scale and Mini Mental State Examination (MMSE) scores, the number of participants with more than one EEG recording and the maximum number of EEGs per participant, the average time between first and last recordings and the time between symptom onset and first EEG are also shown. n: number of participants included in the study. The number of participants excluded due to high amount of artefacts on the EEG are shown in brackets.

IPD, inherited prion disease; NA, not applicable; sCJD, sporadic Creutzfeldt-Jakob disease.

Ninety-six participants had EEG of suitable quality at the NHNN. Twenty-nine participants were healthy controls, and 67 had or were at-risk of prion disease. The demographic details of the participants are shown in table 1. For the purpose of this study, the patient group was divided into three disease groups, containing the participants with inherited prion disease (IPD) with motor or cognitive deficits (symptomatic IPD or sIPD), those that not yet have any symptoms of IPD (asymptomatic or aIPD) and those with sCJD. All IPD patients had a diagnosis confirmed by molecular genetic testing of the *PRNP*. The sCJD patients all fulfilled epidemiological criteria for probable CJD in life, and 13 cases were confirmed by postmortem examination. At *PRNP* codon 129, two sCJD patients were methionine homozygous, three were valine homozygous and nine were heterozygous. The aIPD participants did not have any identifiable symptoms of prion disease during the data collection. The prevalence of the different mutations in the IPD groups were as follows—asymptomatic IPD: P102L:11, A117V:1, E200K:6, D178N:2, 5OPRI:1; 6OPRI:2; symptomatic IPD: P102L:10, A117V:3, E200K:1, D178N:2, 5OPRI:4; 6OPRI:5, Y163X:3, Q212P:1, E196K:1. Three of the 30 sIPD patients were asymptomatic at the time of recruitment and developed symptoms of the disease during the follow-up period. Informed consent was obtained from each participant or from their relatives according to the provisions of the Mental Capacity Act 2005. Ethical approval was obtained from the Scotland A Research Ethics Committee.

### EEG recording and analysis

EEGs were recorded in the EEG laboratory of the NHNN according to the International Federation of Clinical Physiology guidelines (http://www.clinph-journal.com/content/guidelinesIFCN, last accessed 8 February 2015), using a 19-channel Nicolet EEG system with Ag/AgCl surface electrodes. The impedance was kept below 5 kΩ and sampling rate was 256 Hz. The electrodes were placed according to the international 10–20 system. The reference was placed close to electrode Pz. Each recording session lasted for ∼20 min. The data using the common reference were then inspected, and eye movements and high-frequency artefacts were manually removed. Similarly, parts of the recording showing sleep I or II stage were removed.

Further analyses were performed using the EEGLAB (eeglab12_0_2_1b) toolbox of Matlab (MathWorks, USA). Data were divided into 3 s epochs and bandpass filtered using Finite Impulse Response (FIR) between 1 and 25 Hz. Epochs containing artefacts were removed. Artefact rejection was partly manual (based on visual inspection) and automatic (based on thresholding). Additionally, all epochs containing markers (eg, eye open, eye closed) were rejected because they likely contained artefacts (signal of no interest). The percentages of epochs removed due to artefacts in the different groups were the following—control: 6%, aIPD: 10%, sIPD: 20% and sCJD: 29%. We then applied baseline correction on the artefact-free epochs using the first 500 ms of the epoch. We used Independent Component Analysis (ICA) to remove artefacts that might have been left in the epochs after the manual rejection. The frequency content of the epochs between 0 and 128 Hz was computed using fast Fourier transform (FFT) with steps of 0.33 Hz. After averaging the result of the FFT analysis across epochs, we defined the main background frequency as the frequency of the maximum power between 4 and 15 Hz. We also calculated the total power within the α (8–12 Hz) and θ (4–7.6 Hz) bands as the area under the curve and the ratio of α and θ power. Power was defined as the squared amplitude at each frequency and normalised by dividing it with the average power across trials.

**Figure 1:**
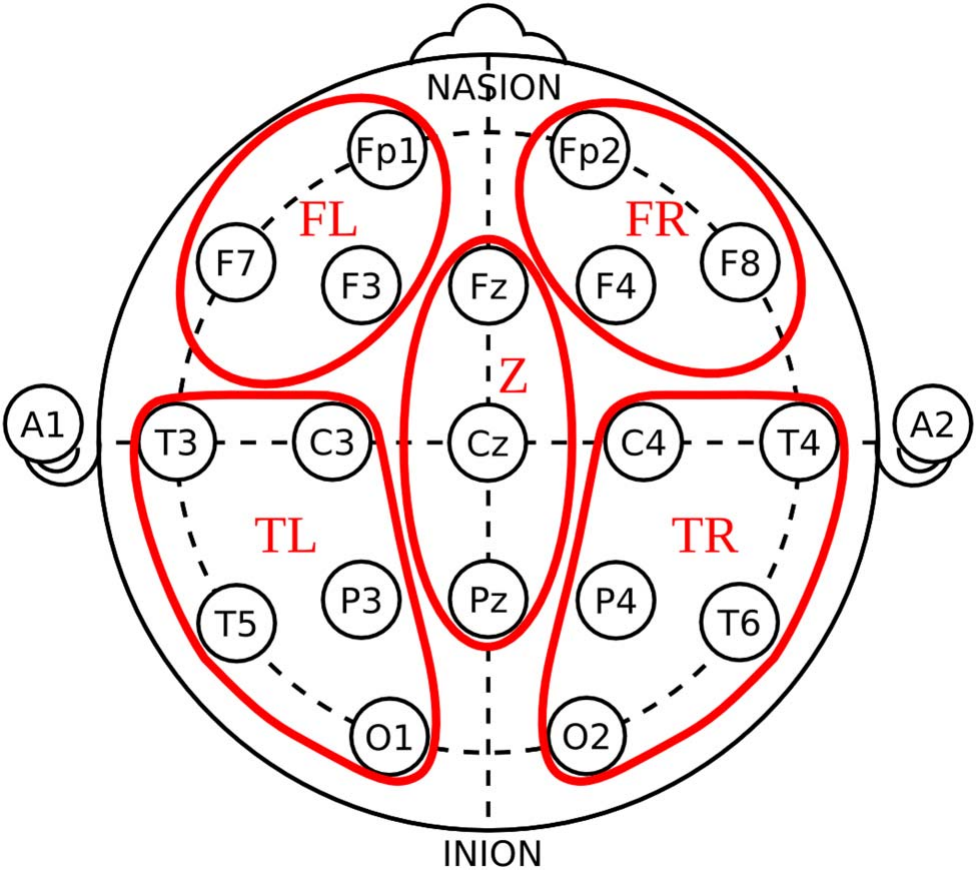
Location and grouping of electrodes.

To reduce the dimensionality of the data, we grouped the electrodes by regions of the scalp. The background frequency and the power spectrum were not used to define the regions, they were simply based on topography. We defined the following five groups: frontal left (FL) and frontal right (FR) electrodes, including FL: Fp1, F3, F7; FR: Fp2, F4, F8; central (Z) electrodes, including Z: Fz, Cz, Pz; and left (TL) and right temporo-occipital (TR) electrodes, including TL: C3, P3, T3, T5, O1; TR: C4, P4, T4, T6, O2 (figure 1).

The main background frequency, the total power in α and θ bands and the power ratio were correlated with the MRC Scale and the MMSE, cross-sectionally and longitudinally. The EEG parameters of the different participant groups were compared cross-sectionally, using the last available EEG recording. Longitudinal analysis was performed using the difference between the first and last EEGs in participants who underwent more than one recording. When comparing the general relationship between the EEG parameters and the clinical scores, we included all available measurements from the patients showing signs of prion disease, namely the sCJD and sIPD groups. The participants in the control and aIPD group all had maximal MRC Scale score and normal range MMSE. In the longitudinal correlation analysis between the EEG parameters and the clinical scores, the difference between the first and last measurements from the symptomatic patients was used.

### Statistics

We used two-tailed t-test to look for significant difference among the participant groups. To control for the effect of MRC Scale score in the comparison between sIPD and sCJD patients, we performed an analysis of covariance with MRC Scale score as a covariate. A paired t-Test was used to check the difference between first and last EEGs for participants with more than one EEG recording. Pearson's correlation was applied to reveal the relationship between EEG parameters and functional and cognitive scores. The significance level was corrected for multiple comparisons using the Bonferroni correction method.

## Results

**Figure 2:**
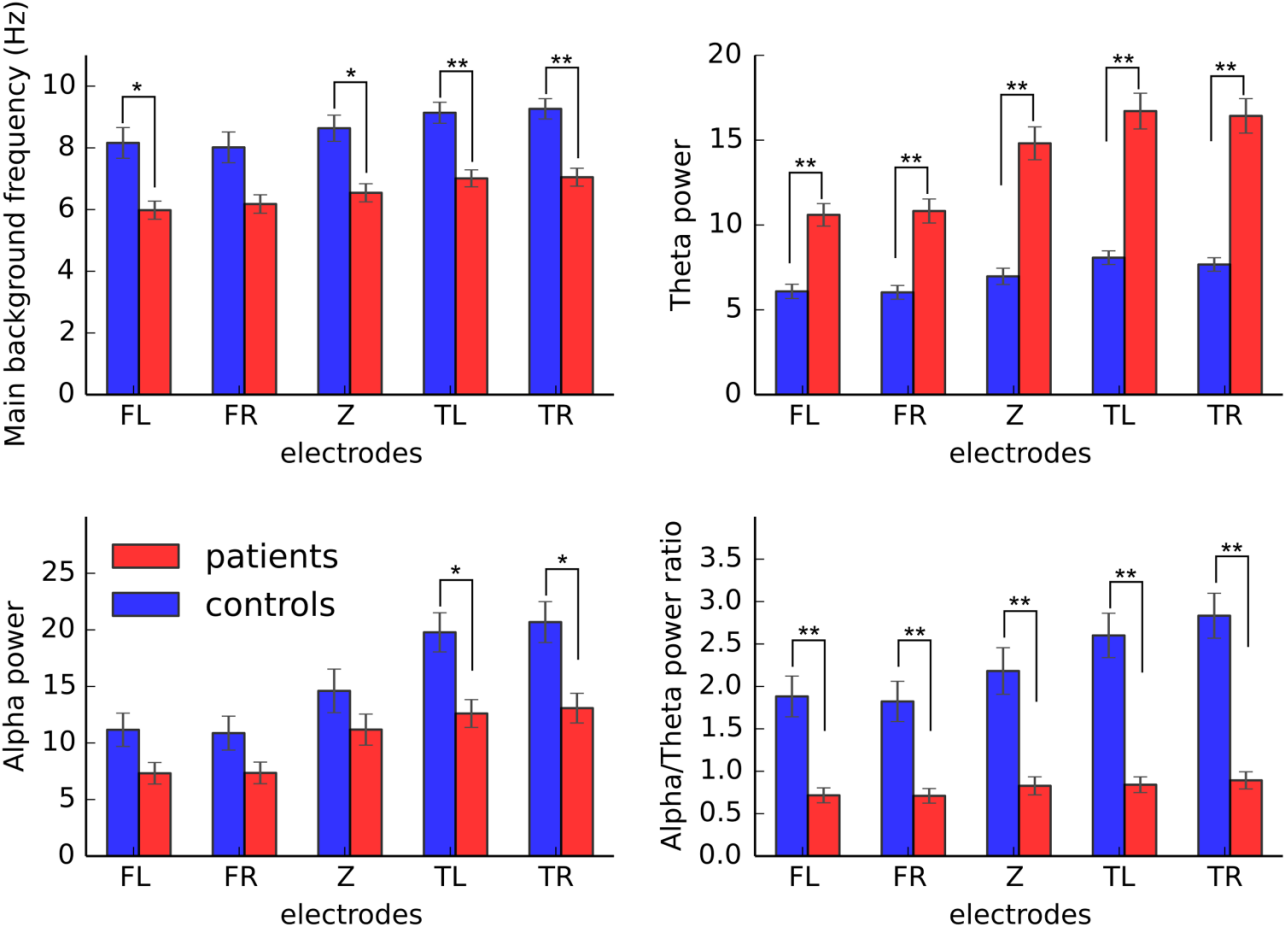
Comparison of EEG parameters between healthy participants and patients with prion disease on the five-electrode groups. Error bars indicate the SEM. *p<0.0025, **p<0.0001. FL, left frontal; FR, right frontal; TL, left temporal; TR, right temporal electrode group; Z, central.

The comparison between control participants (n=29) and symptomatic patients (sIPD and sCJD; n=44) showed highly significant differences in the main background frequency, the θ power and the α/θ power ratio (figure 2, table 2 and see online supplementary table S1).

**Table 2:** p Values for the comparisons between the participant groups for five-electrode groups

	Main background frequency	θ power	α/θ power ratio	α power
Controls vs (sIPD+sCJD)				
FL	4.4×10^−4^	2.4×10^−7^	5.9×10^−5^	ns
FR	ns	1.5×10^−7^	9.2×10^−5^	ns
Z	1.6×10^−4^	8.3×10^−10^	5.0×10^−5^	ns
TL	8.6×10^−6^	3.1×10^−10^	2.7×10^−7^	1.3×10^−3^
TR	3.8×10^−6^	7.8×10^−11^	5.1×10^−8^	1.2×10^−3^
sIPD vs sCJD				
FL	ns	ns	9.4×10^−4^	ns
FR	1.2×10^−4^	ns	1.3×10^−3^	ns
Z	5.6×10^−4^	ns	ns	1.8×10^−3^
TL	4.3×10^−4^	ns	1.5×10^−5^	1.2×10^−5^
TR	1.4×10^−3^	ns	6.6×10^−5^	4.1×10^−5^
aIPD vs sIPD				
FL	3.6×10^−4^	1.5×10^−3^	7.8×10^−5^	ns
FR	1.1×10^−3^	1.0×10^−3^	6.9×10^−5^	ns
Z	1.1×10^−4^	9.1×10^−6^	1.3×10^−5^	ns
TL	2.3×10^−3^	3.8×10^−7^	6.4×10^−7^	1.0×10^−4^
TR	3.8×10^−4^	1.7×10^−7^	9.4×10^−8^	1.4×10^−4^

The significance threshold was adjusted by Bonferroni correction.

FL, left frontal; FR, right frontal; IPD, inherited prion disease; ns, non-significant; sCJD, sporadic Creutzfeldt-Jakob disease; TL, left temporal; TR, right temporal electrode group; Z, central.

10.1136/jnnp-2016-313501.supp1 supplementary table

**Figure 3:**
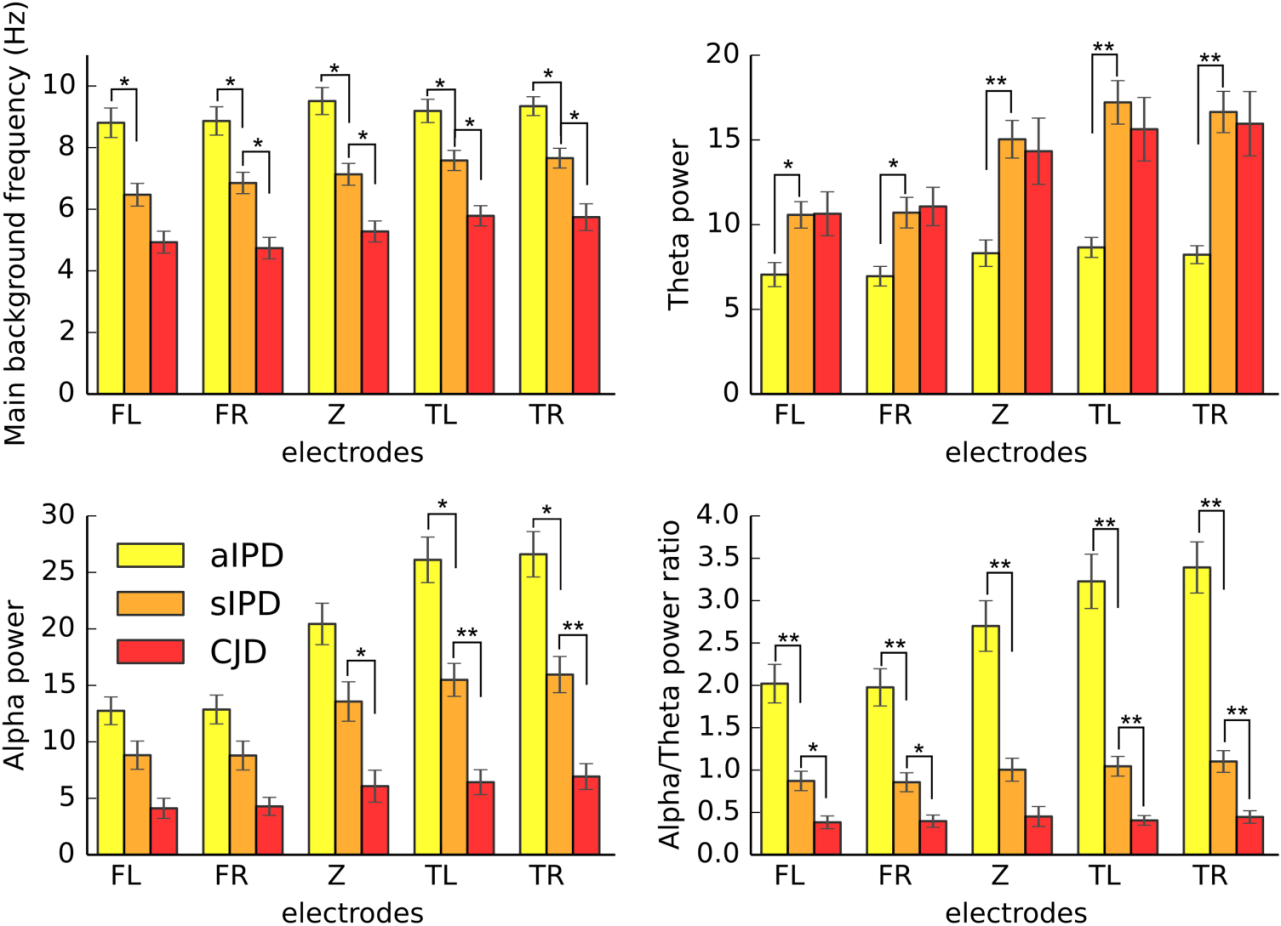
Comparison of EEG parameters among patients with asymptomatic or symptomatic IPD and sCJD on the five-electrode groups. Error bars indicate the SEM. *p<0.0025, **p<0.0001. FL, left frontal; FR, right frontal; IPD, inherited prion disease; sCJD, sporadic Creutzfeldt-Jakob disease; TL, left temporal; TR, right temporal electrode group; Z, central.

As expected, the main frequency was lower in patients than in controls, whereas they had increased power in the θ band and decreased α/θ power ratio. On the temporo-occipital electrodes, the α power could also distinguish between these two groups, being lower in patients than in controls. We next subdivided the patient group to evaluate whether there were differences among the patients, namely the sCJD (n=14) and asymptomatic (n=23) and symptomatic IPD (n=30) patients (figure 3). These comparisons revealed that patients with sCJD showed similar direction of parameter change as symptomatic IPD; however, they had significantly lower main background frequency, α power and α/θ power ratio on most of the electrode groups (except for FL for peak location and FR for α power), consistent with the more advanced clinical progression of the sCJD group (see online supplementary table S1 for p values after using MRC Scale as covariate). The symptomatic IPD patients showed significantly lower main background frequency than the asymptomatic carriers on all the electrode groups. They also had higher θ power and lower α/θ power ratio all over the scalp. The α power difference reached significant level between the aIPD and sIPD patients only on the temporal electrode groups. The asymptomatic IPD patients did not differ significantly from the control group in any of these measurements. For all the significance values among patient groups, see online supplementary table S1.

**Figure 4:**
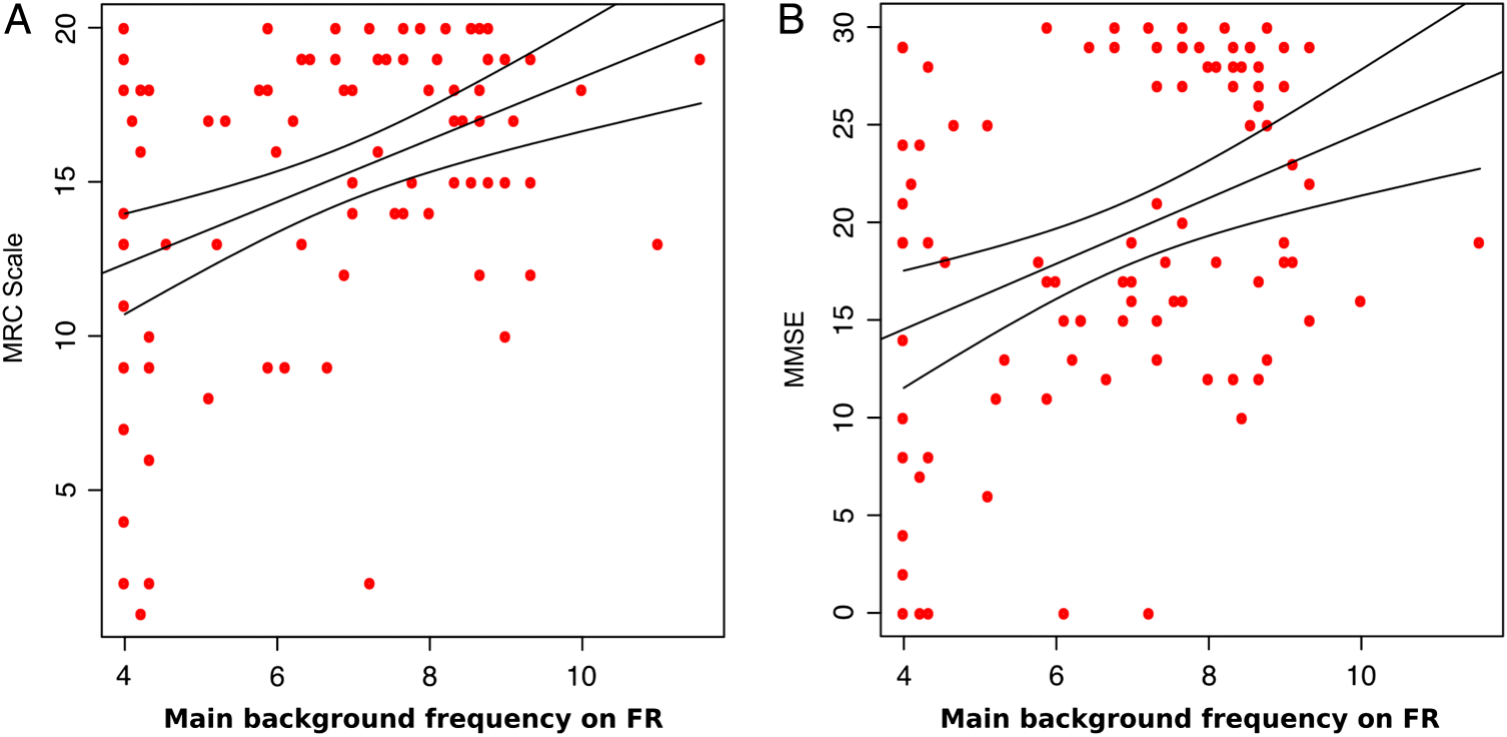
Correlation between the main background frequency and the MRC Scale (A)/ Mini Mental State Examination (MMSE) (B) scores for the symptomatic patients (sIPD and sCJD) with the 95% CI on the right frontal electrode group. FR, right frontal.

Further, we examined whether qEEG parameters reflect the differences in the extent of disease progression in patients (figure 4). We correlated all available cross-sectional data from sCJD and symptomatic IPD patients with the MRC Scale score and the MMSE. We found that the main background frequency and the α/θ power ratio correlated significantly with the MRC Scale on most of the electrode groups, namely being lower with worsening score. The α power also reflected the worsening on the left and right temporo-occipital electrodes (table 3).

**Table 3:** Correlation coefficients (r) and p values for the significant correlations between MRC Scale and EEG parameters

MRC Scale						
	Main background frequency		α/θ power ratio		α power	
	r	p Value	r	p Value	r	p Value
FL	0.39	9.0×10^−5^	0.33	1.1×10^−3^	0.29	ns
FR	0.40	5.7×10^−5^	0.31	2.1×10^−3^	0.28	ns
Z	0.35	4.3×10^−4^	0.26	ns	0.28	ns
TL	0.37	2.4×10^−4^	0.35	3.9×10^−4^	0.42	1.7×10^−5^
TR	0.43	1.3×10^−5^	0.34	6.6×10^−4^	0.41	3.6×10^−5^

The significance threshold was adjusted by Bonferroni correction.

FL, left frontal; FR, right frontal; ns, non-significant; TL, left temporal; TR, right temporal electrode group; Z, central.

Significant correlation between the MMSE and the main background frequency was found on the frontal and central electrodes. Similarly to the MRC Scale, the correlation was positive, showing lower background frequency in patients with lower MMSE score. Significant positive correlation was found with the α/θ ratio on the frontal and the right temporo-occipital electrodes. Correlation coefficients and p values are shown in table 3 for the MRC Scale and in table 4 for the MMSE and in online supplementary table S1.

**Table 4:** Correlation coefficients (r) and p values for the significant correlations between MMSE and EEG parameters

MMSE						
	Main background frequency		α/θ power ratio		α power	
	r	p Value	r	p Value	r	p Value
FL	0.37	2.0×10^−4^	0.35	4.2×10^−4^	0.24	ns
FR	0.36	2.8×10^−4^	0.34	7.7×10^−4^	0.25	ns
Z	0.32	1.4×10^−3^	0.26	ns	0.20	ns
TL	0.25	ns	0.28	ns	0.22	ns
TR	0.29	ns	0.31	1.6×10^−3^	0.25	ns

The significance threshold was adjusted by Bonferroni correction.

FL, left frontal; FR, right frontal; MMSE, Mini Mental State Examination; ns, non-significant; TL, left temporal; TR, right temporal electrode group; Z, central.

**Figure 5:**
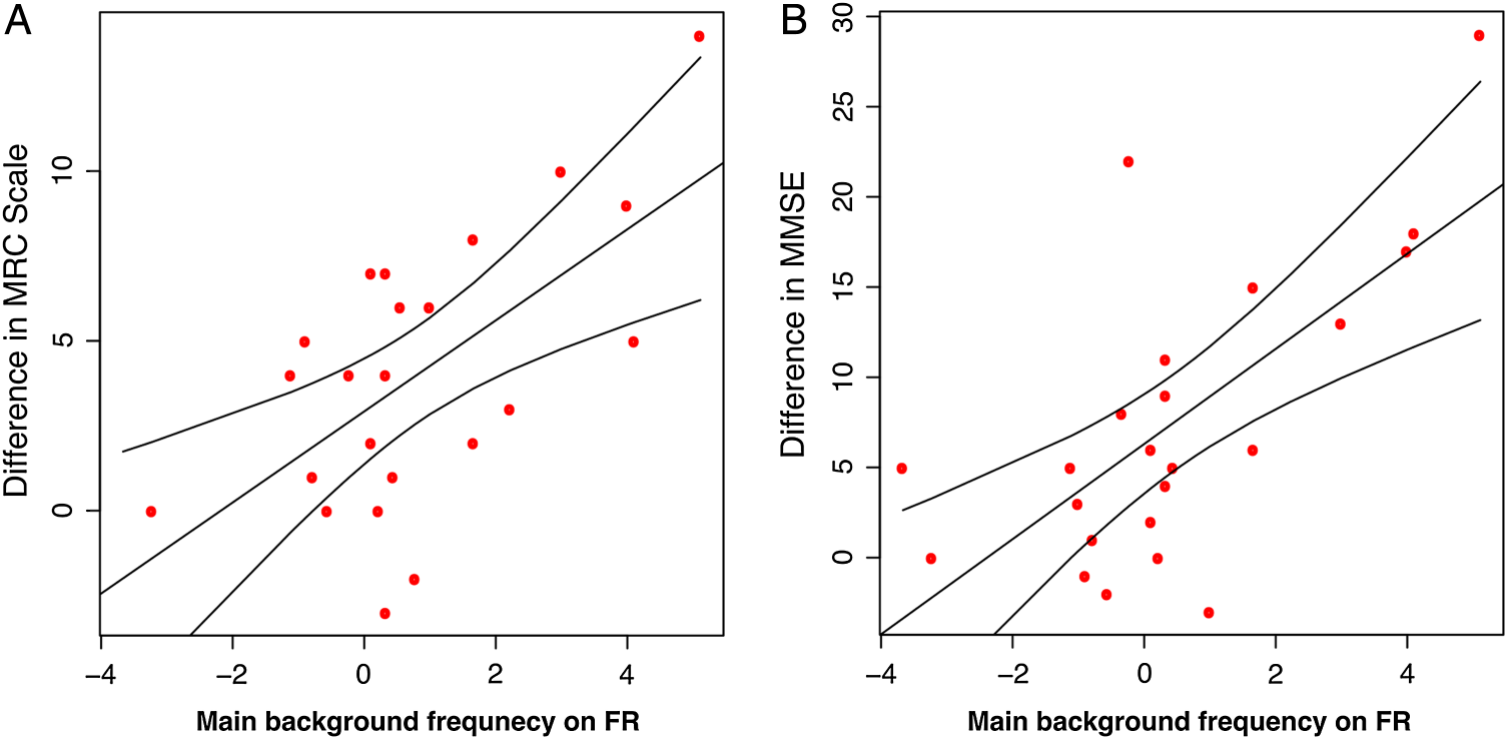
Correlation between the change in main background frequency and the change in MRC Scale (A)/Mini Mental State Examination (MMSE) (B) scores over time for the symptomatic patients (sIPD and sCJD) with the 95% CI on the right frontal electrode group. FR, right frontal.

Finally, we found significant longitudinal correlation between change in qEEG parameters and the change in clinical scores over time (figure 5). Table 1 details the number of EEGs performed, the time interval and numbers of patients. Worsening in MRC Scale score correlated significantly with the decrease in the main background frequency on FR (r=0.62, p=1.5×10^−3^) but showed a trend, in all the other electrode groups (p<0.05). A trend of correlation with the decrease in α power was also observed on the temporal electrodes (p<0.05). Similarly, the correlation with the MMSE was significant on FR with the main background frequency (r=0.67, p=3.8×10^−4^) and on TR with the α/θ ratio (r=0.60, p=2.3×10^−3^). However, it showed a trend (p<0.05) on all electrodes with the background frequency and α/θ ratio, and on the central and temporo-occipital electrodes with the α power. All p values are shown in online supplementary table S1.

## Discussion

We aimed to develop qEEG biomarkers of disease progression in prion diseases with potential utility in an experimental medical setting. There are a number of reasons to expect prion diseases to be peculiarly appropriate for this technology. Pathological features of prion disease are widespread throughout the cerebral cortex, subcortical nuclei, brainstem and cerebellum, meaning that biomarkers of brain structure are unlikely to be as useful as they are in neurodegenerative diseases associated with focal atrophy. Furthermore, EEG PSWCs have long been recognised as a marked neurophysiological feature and incorporated into epidemiological diagnostic criteria. However, these characteristic waves are more frequent in certain types of sCJD (MM1 and MV1)^9^ ^20^ patients, whereas they rarely occur in VV2 and MV2 types.^9^ ^21^ Our goal was to develop a biomarker that is representative of all types of prion diseases (the different sCJD subtypes and the inherited forms). To this end, we used for the first time qEEG analysis and evaluated the difference in qEEG parameters between patients with different forms of prion diseases and healthy controls.

Our analyses revealed highly significant decreases in the main background frequency, α power and α/θ power ratio and significant increase in θ power in patients compared to healthy participants. These findings are in accordance with studies comparing patients with Alzheimer's disease, mild cognitive impairment and healthy elderly controls.^16^ ^22–24^ Similarly, patients with Parkinson's disease and cognitive decline have increased θ and decreased α power and significantly lower median frequency compared to healthy controls.^25^ ^26^ When subdividing the patient group, we found lower background frequency, α power and α/θ power ratio in patients with sCJD when compared to the IPD group, in parallel to differences in the MRC Scale scores. Asymptomatic carriers of prion protein gene mutation did not show any EEG abnormalities.

To evaluate these EEG differences as potential progression biomarkers, we correlated the derived EEG parameters with the clinical and cognitive scores. We found that the main background frequency and the α/θ power ratio correlated significantly with the MRC Scale score and MMSE; namely that patients with lower clinical and cognitive scores had lower main frequency and α/θ power ratio. We also found significant correlation between the α power and the MRC Scale score. Similarly to our findings, previous studies have also described lower peak frequency and lower α activity during memory tasks in patients with Alzheimer's disease who had decreased MMSE or Global Deterioration Scale scores or decline on the Cambridge Cognitive Examination.^17^ ^24^ ^27^ ^28^ Besides the significant correlation in the cross-sectional data, we found that the decrease in main background frequency over time also showed correlation with the functional and cognitive decline in prion patients. The α power and α/θ power ratio also decreased with decrease in MRC and MMSE scores. Similarly to our findings, others have reported reduced α power after 1-year follow-up in patients with progressive mild cognitive impairment and Alzheimer's disease when compared to those with stable mild cognitive impairment.^29^ Another longitudinal study examining patients with Parkinson's disease and cognitive decline showed significant correlation between the increase in delta power and the reduction in MMSE score. Even though the change in background frequency, θ and α power did not show correlation with the decrease in MMSE in their study, a more sensitive neuropsychological test (Auditory Verbal Learning Test) correlated significantly with the change in background frequency.^30^

Our studies of a rare disease are necessarily limited by sample size. In the longitudinal analysis, we had less data than in the cross-sectional tests, because we could only include those participants who had at least two good-quality EEGs. The smaller sample size, therefore, be responsible for variability in the estimated correlations between the EEG parameters and the functional and cognitive scores. Nevertheless, the strong effects we detected show that these EEG measurements are able to reflect the progression of the disease within patients with prion diseases. Taken together with the case–control findings, these data suggest that the predictive or longitudinal changes in qEEG parameters might be a shared consequence of neurodegeneration in different types of dementias.

Artefacts decreased the quality of many EEG recordings and resulted in removal of part or the entire recording. The most common type of artefacts was due to eye movements. This included blinks and the movements of the eyeballs. Parts of the EEG recording containing any eye movement artefacts were rejected either automatically by independent component analysis or manually after inspection. Recording the electro-oculogram, which was not possible in the current study, would make the removal of these artefacts more precise in the future. Drowsiness and sleep during the EEG recording also resulted in rejection of part of the data. The normal decrease in background frequency during sleep could mimic the abnormal decrease in the main frequency, which is the result of the disease. Therefore, all parts of the recordings that showed signs of sleep I or II stages were manually rejected. Avoiding the participants sleeping by intermittent interaction during the recording could reduce this type of artefact. A small proportion of data had to be rejected because of high-frequency artefact or low-amplitude signal that could not be eliminated during the recording session.

The current study was also limited by the relatively small number of sCJD patients. At present in the UK, sCJD is often diagnosed late in the clinical course when patients are too unwell to travel for research neurophysiological studies. It was also difficult to record more than one EEG in sCJD because of the rapid progression of the disease. The use of different recording systems and different file types made difficult to use EEGs recorded from other hospitals, and we therefore decided to focus on the most homogeneous subset recorded at NHNN. If the qEEG parameters we report here were to be used in an exploratory fashion to provide supportive evidence for alteration in the natural history of sCJD in trials of experimental therapeutics, then enrolment rates would likely be substantially higher.

We did not find any differences between qEEG parameters in asymptomatic gene mutation carriers compared with healthy controls. Two interpretations are plausible: (1) the EEG does become abnormal several years before clinical onset, reflecting incipient neurodegeneration, but there were too few patients close to actual clinical onset in the aIPD group to detect this and (2) the EEG only becomes abnormal in IPD at clinical onset. Continued follow-up of aIPD patients and retrospective analysis of converting clinical cases may be helpful.

In conclusion, we found strong correlations between several qEEG parameters and prion disease diagnosis, severity and progression over time. Priorities for future work include the use of these technologies in a clinical trial setting as an exploratory biomarker, the continued study of healthy at-risk individuals and consideration of related technologies such as magnetoencephalography.
