# Supplementary material for: Quantitative EEG parameters correlate with the progression of human prion diseases
Source: J Neurol Neurosurg Psychiatry. 2016 Jul 13;87(10):1061–7. doi: 10.1136/jnnp-2016-313501 (PMC5036210; doi:10.1136/jnnp-2016-313501)
Supplement: supplementary table [file jnnp-2016-313501supp_table.pdf]

		P-value				
Main background frequency		FL	FR	Z	TL	TR
	controls vs patients	4.49E-004	0.002778	1.61E-004	8.67E-006	3.89E-006
	controls vs aIPD	0.3545	0.2168	0.1574	0.9169	0.8581
	controls vs sIPD	0.008584	0.06132	0.008688	0.001674	0.0009262
	controls vs sCJD	4.66E-006	3.26E-006	2.69E-007	2.35E-008	5.21E-007
	aIPD vs sIPD	3.61E-004	0.001116	1.17E-004	0.002331	3.80E-004
	aIPD vs sCJD	1.80E-007	2.49E-008	6.23E-009	7.53E-008	3.70E-007
	sIPD vs sCJD	0.004775	1.27E-004	5.65E-004	4.33E-004	0.001414
	sIPD vs sCJD/MRCS covariate	0.04306	0.004966	0.002402	0.002433	0.002168
Theta power		FL	FR	Z	TL	TR
	controls vs patients	2.41E-007	1.57E-007	8.39E-010	3.17E-010	7.86E-011
	controls vs aIPD	0.2508	0.1963	0.1525	0.4204	0.4064
	controls vs sIPD	7.40E-006	2.88E-005	5.88E-008	7.43E-008	3.75E-008
	controls vs sCJD	0.00415	6.55E-004	0.002442	0.001426	7.67E-004
	aIPD vs sIPD	0.001539	0.001052	9.18E-006	3.85E-007	1.73E-007
	aIPD vs sCJD	0.02401	0.004173	0.01078	0.002738	0.001366
	sIPD vs sCJD	0.9653	0.8033	0.7567	0.4919	0.761
	sIPD vs sCJD/MRCS covariate	0.6584	0.8601	0.9246	0.8748	0.9743
Alpha power		FL	FR	Z	TL	TR
	controls vs patients	0.03303	0.05401	0.1535	0.001338	0.001239
	controls vs aIPD	0.4126	0.3172	0.03303	0.02132	0.03344
	controls vs sIPD	0.2295	0.2955	0.6915	0.06305	0.05512
	controls vs sCJD	1.95E-004	4.02E-004	9.41E-004	8.19E-008	1.08E-007
	aIPD vs sIPD	0.02924	0.02846	0.009175	1.09E-004	1.47E-004
	aIPD vs sCJD	1.95E-006	2.19E-006	4.31E-007	8.17E-010	8.21E-010
	sIPD vs sCJD	0.00383	0.004694	0.001849	1.28E-005	4.10E-005
	sIPD vs sCJD/MRCS covariate	0.04442	0.07661	0.01739	0.002434	0.010545
Alpha/theta power ratio		FL	FR	Z	TL	TR
	controls vs patients	5.91E-005	9.20E-005	5.01E-005	2.71E-007	5.09E-008
	controls vs aIPD	0.6783	0.6368	0.2071	0.1366	0.171
	controls vs sIPD	4.88E-004	6.82E-004	4.12E-004	3.20E-006	6.56E-007
	controls vs sCJD	1.10E-006	2.06E-006	1.26E-006	3.22E-009	6.26E-010
	aIPD vs sIPD	7.89E-005	6.95E-005	1.31E-005	6.43E-007	9.45E-008
	aIPD vs sCJD	2.71E-007	2.69E-007	1.23E-007	9.17E-009	1.12E-009
	sIPD vs sCJD	9.45E-004	0.001324	0.003839	1.49E-005	6.64E-005
	sIPD vs sCJD/MRCS covariate	0.01538	0.02175	0.01018	0.00336	0.008549

MRC score and EEG correlation
(all data from sIPD+sCJD)

		FL		FR		Z		TL		TR	
		P-value	r	P-value	r	P-value	r	P-value	r	P-value	r
Main background frequency	controls vs patients	9.00E-005	0.39	6.00E-005	0.4	4.00E-004	0.35	2.45E-004	0.37	1.30E-005	0.43
Theta power	controls vs patients	0.6648	-0.04	0.232	-0.12	0.4176	-0.08	0.6818	-0.04	0.3715	-0.09
Alpha power	controls vs patients	0.0044	0.29	0.006472	0.28	0.006551	0.28	1.77E-005	0.42	3.65E-005	0.41
Alpha/theta power ratio	controls vs patients	0.001108	0.33	0.00217	0.31	0.009107	0.26	3.94E-004	0.35	6.63E-004	0.34

MMSE and EEG correlation
(all data from sIPD+sCJD)

		FL		FR		Z		TL		TR	
		P-value	r	P-value	r	P-value	r	P-value	r	P-value	r
Main background frequency	controls vs patients	2.00E-004	0.37	3.00E-004	0.36	0.001495	0.32	0.01377	0.25	0.004029	0.29
Theta power	controls vs patients	0.7848	-0.02	0.6127	-0.05	0.3165	-0.1	0.4679	-0.07	0.4303	-0.08
Alpha power	controls vs patients	0.01704	0.24	0.01216	0.25	0.04621	0.2	0.02885	0.22	0.01247	0.25
Alpha/theta power ratio	controls vs patients	4.00E-004	0.35	8.00E-004	0.34	0.01126	0.26	0.004967	0.28	0.001699	0.31

Correlation of change in MRC score and EEG over time
(first-last: sIPD+sCJD)

		FL		FR		Z		TL		TR	
		P-value	r	P-value	r	P-value	r	P-value	r	P-value	r
Main background frequency	controls vs patients	0.04127	0.42	0.001556	0.62	0.01269	0.51	0.03889	0.43	0.02508	0.46
Theta power	controls vs patients	0.9966	0	0.7397	-0.07	0.9993	0	0.6019	-0.11	0.2735	-0.23
Alpha power	controls vs patients	0.3388	0.2	0.2078	0.27	0.1202	0.33	0.03159	0.44	0.007043	0.54
Alpha/theta power ratio	controls vs patients	0.2127	0.27	0.1223	0.33	0.3471	0.2	0.1094	0.34	0.02312	0.47

Correlation of change in MMSE and EEG over time
(first-last: sIPD+sCJD)

		FL		FR		Z		TL		TR	
		P-value	r	P-value	r	P-value	r	P-value	r	P-value	r
Main background frequency	controls vs patients	0.002911	0.59	4.00E-004	0.67	0.01033	0.52	0.01408	0.5	0.012	0.51
Theta power	controls vs patients	0.5675	-0.12	0.3207	-0.21	0.5694	-0.12	0.307	-0.22	0.133	-0.32
Alpha power	controls vs patients	0.0818	0.37	0.06969	0.38	0.04911	0.41	0.01678	0.49	0.004522	0.56
Alpha/theta power ratio	controls vs patients	0.01368	0.5	0.00904	0.53	0.04543	0.42	0.01816	0.48	0.002361	0.6
